# Supplementary material for: The Evolution and Characterization of the RNA Interference Pathways in Lophotrochozoa
Source: Genome Biol Evol. 2024 May 7;16(5):evae098. doi: 10.1093/gbe/evae098 (PMC11114477; doi:10.1093/gbe/evae098)
Supplement: evae098_Supplementary_Data [file evae098_supplementary_data.zip › Supplementary_materials.zip!/selected_miRNAs/NoGe_selected_miRNAs/mirdeep_pdf_files/NMRB01000185.1_Notospermus_geniculatus_strain_Ushimado_scaffold185,_whole_genome_shotgun_sequence_6740.pdf]

Provisional ID : NMRB01000185.1_Notospermus_geniculatus_strain_Ushimado_scaffold185,_whole_genome_shotgun_sequence_6740
Score total : 351.1
Score for star read(s) : 3.9
Score for read counts : 347.3
Score for mfe : 0.5
Score for randfold : 0
Score for cons. seed : -0.6
Total read count : 693
Mature read count : 381
Loop read count : 0
Star read count : 312

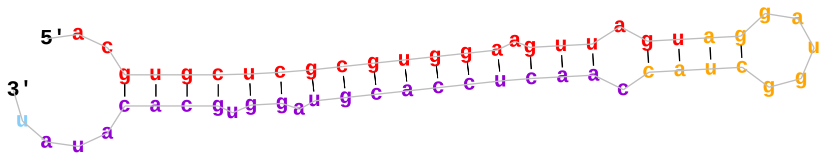

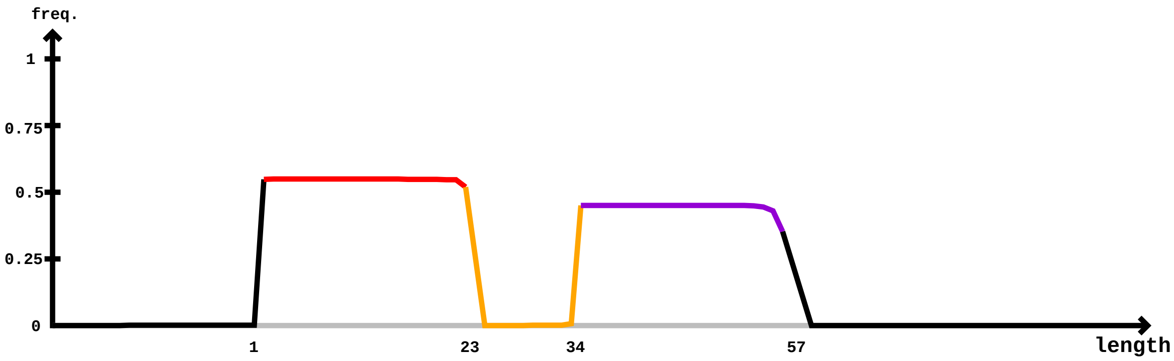

Mature Star

```
5'- ccggacuccaguagucaaucacgugcucgcguggaaguuaguaggauggcuaccaacuccacguaggugcacauauuaagaaucaagauuaggauuuuuuuucgauagacag  -3'  obs
    ccggacuccaguagucaaucacgugcucgcguggaaguuaguaggauggcuaccaacuccacguaggugcacauauuaagaaucaagauuaggauuuuuuuucgauagacag       exp
    ............(((.(((..((((((((((((.(((.((((.....)))).))))))))))).)).)))).....(((((((........)))))))....))).)))..  reads  mm  sample
    ....................acgugcucgcguggaaguu.........................................................................  1  0  Mm3
    ....................acgugcucgcguggaaguuUgu......................................................................  24  1  Mm3
    ....................acgugcucgcguggaaguuUgua.....................................................................  3  1  Mm3
    .....................................................Aacuccacguaggugcacau.......................................  2  1  Mm3

    ....................acgugcucgcguggaaguuUg.......................................................................  19  1  Mm1
    ....................acgugcucgcguggaagCuagu......................................................................  1  1  Mm1
    ....................acgugcucgcguggaaguuUgu......................................................................  51  1  Mm1
    ....................acgugcuAgcguggaaguuagu......................................................................  1  1  Mm1
    ....................acgugcucgcguggaaguuGgu......................................................................  10  1  Mm1
    ....................acgugcucgcguggaaUuuagu......................................................................  1  1  Mm1
    ....................acgugcucgcguggaagGuagu......................................................................  3  1  Mm1
    ....................acgugcucgcguggaaguuagu......................................................................  82  0  Mm1
    ....................acgugcucgcguggaaguuaguU.....................................................................  10  1  Mm1
    ....................acgugcucgcguggaaguuUgua.....................................................................  1  1  Mm1
    .....................................................caacuccacguaggugcacauU.....................................  1  1  Mm1
    .....................................................caacuccacguaggugcacaua.....................................  5  0  Mm1

    ......uccaguagucaaucacgGcucgcgugga..............................................................................  1  1  Mm2
    ....................acgugcucgcguggaagCuagu......................................................................  1  1  Mm2
    ....................acgugcucgcguggaaguuUgu......................................................................  52  1  Mm2
    ....................acgugcucgcguggaaguuagu......................................................................  41  0  Mm2
    ....................acgugcucgcguggaaguuaguU.....................................................................  3  1  Mm2
    ....................acgugcucgcguggaaguuagua.....................................................................  1  0  Mm2
    .....................cgugcucgcguggaaguuUgu......................................................................  1  1  Mm2
    ....................................................ccaacuccacguaggugcacau......................................  1  0  Mm2
    ....................................................cAaacuccacguaggugcacau......................................  2  1  Mm2
    ....................................................ccaacuccacguaggugcacaua.....................................  1  0  Mm2
    .....................................................caacuccacguaggugcaca.......................................  2  0  Mm2
    .....................................................Aacuccacguaggugcacau.......................................  3  1  Mm2
    .....................................................caacuccacguaggugcacau......................................  7  0  Mm2
    .....................................................caacuccacguaggugcacauU.....................................  6  1  Mm2
    .....................................................caacuccacguaggugcacaua.....................................  17  0  Mm2
```

```
                    Mature                           Star
ccggacuccaguagucaaucacgugcucgcguggaaguuaguaggauggcuaccaacuccacguaggugcacauauuaagaaucaagauuaggauuuuuuuucgauagacag
.....................................................caacuccacguaggugcacauUu....................................    5    1    Mm2
.....................................................caacuccacguaggugcacauau....................................    33   0    Mm2
.....................................................Aaacuccacguaggugcacauau....................................    1    1    Mm2
.....................................................caacuccacguaggugcacauauu...................................    15   0    Mm2
.....................................................caacuccacguaggugcacauaAu...................................    2    1    Mm2

....................acgugcucgcguggaaguuUgu......................................................................    36   1    Fm3
....................acgugcucgcguggaaguuUgua.....................................................................    2    1    Fm3

....................acgugcucgcguggaaguuagu......................................................................    6    0    Fm2
....................acgugcucgcguggaaguuUgu......................................................................    9    1    Fm2
.....................................................caacuccacguaggugcacauU.....................................    7    1    Fm2

....................acgugcucgcguggaaguuUgu......................................................................    13   1    Fm1
....................Ncgugcucgcguggaaguuagu......................................................................    1    1    Fm1
....................acgugcucgcguggaaguuagu......................................................................    8    0    Fm1
................................................gcuacAaacuccacguaggugca.........................................    1    1    Fm1
.....................................................Aaacuccacguaggugcac........................................    3    1    Fm1
.....................................................caacuccacguaggugcaca.......................................    3    0    Fm1
.....................................................Aaacuccacguaggugcaca.......................................    5    1    Fm1
.....................................................caacuccacguaggugcacau......................................    37   0    Fm1
.....................................................Aaacuccacguaggugcacau......................................    2    1    Fm1
.....................................................Aaacuccacguaggugcacaua.....................................    5    1    Fm1
.....................................................caacuccacguaggugcacaua.....................................    36   0    Fm1
.....................................................caacuccacguaggugcacauU.....................................    23   1    Fm1
.....................................................caacuccacguaggugcUcaua.....................................    1    1    Fm1
.....................................................caacuccacgGaggugcacaua.....................................    1    1    Fm1
.....................................................Aaacuccacguaggugcacauau....................................    7    1    Fm1
.....................................................caacuccacguaggugcacauUu....................................    5    1    Fm1
.....................................................caacuccacguaggugAacauau....................................    1    1    Fm1
.....................................................caacuccacguaggugcacauau....................................    49   0    Fm1
.....................................................caacuccacguaggugcCcauauu...................................    1    1    Fm1
.....................................................caacuccacguaggugcacauaCu...................................    1    1    Fm1
.....................................................caacuccacguaggugcacauauu...................................    22   0    Fm1
```
